# Magnetic Resonance Imaging in Children under Anesthesia: The Relationship between the Degree of Information Provided to Parents and Parents' Anxiety Scores

**DOI:** 10.1155/2014/425107

**Published:** 2014-05-25

**Authors:** Abdulmenap Güzel, Abdullah Atlı, Erdal Doğan, Feyzi Çelik, Adnan Tüfek, Abdurrahim Dusak, Velat Şen, Zeynep Baysal Yıldırım

**Affiliations:** ^1^Department of Anesthesiology and Reanimation, Faculty of Medicine, Dicle University, 21280 Diyarbakır, Turkey; ^2^Department of Psychiatry, Faculty of Medicine, Dicle University, 21280 Diyarbakır, Turkey; ^3^Department of Radiology, Faculty of Medicine, Dicle University, 21280 Diyarbakır, Turkey; ^4^Department of Pediatrics, Faculty of Medicine, Dicle University, 21280 Diyarbakır, Turkey

## Abstract

*Background*. We aimed to investigate the correlation between the anxiety scores of parents whose children are administered anesthesia for magnetic resonance imaging (MRI) and the level of information provided to them before the MRI. The study included 146 children and their parents. The demographic information of the children and their parents was recorded. The parents were divided into two groups. In Group I, the patient's medical history and physical exam findings were recorded on a standard consultation form by an anesthesiologist. In Group II, the parents were additionally provided with more detailed information on how the anesthesia would be administered and the drugs to be used and their side effects and complications. The anxiety scores of the parents were found to be lower in Group II. A higher level of education was associated with higher anxiety scores. Intergroup comparison detected lower anxiety scores for Group II parents whose education levels were up to high school. However, no change upon receiving detailed information was detected in the anxiety scores of parents with higher education levels. In conclusion, this may lower the anxiety scores in parents informed about details of anesthesia administration and may raise parents' sense of confidence in the doctor.

## 1. Introduction

Magnetic resonance imaging (MRI) is an imaging technique that has been increasingly used because it provides high quality imaging and does not use ionizing radiation. Since MRI requires complete stillness, images are often obtained under deep sedation or general anesthesia, especially if the patient is a child [1–6]. Additionally, factors like the closed MRI working environment, noises during the scan, and the presence of unknown medical staff cause agitation and restlessness in children, as well as intense anxiety in their parents [7–9]. A significant relationship between the anxiety scores of children undergoing MRI and those of their parents has been demonstrated [10, 11]. Thus, MRI units should include in their routines standard monitoring of the child under anesthesia and elimination of parental anxiety. Explaining MRI to parents is important in decreasing parental anxiety, but the level of information that should be provided is still a matter of dispute [10–16].

The purpose of this study was to assess the relationship between the anxiety scores of parents whose children underwent anesthesia for MRI and the amount of information provided to the parents prior to the MRI test.

## 2. Materials and Methods

The study plan was approved by the Ethics Committee of the Medical School Hospital of Dicle University, Diyarbakır, Turkey. The study included 146 children under 10 years who underwent diagnostic MRI under anesthesia and their parents. Each child's age, gender, weight, ASA score, and body part imaged were noted, along with the duration of the MRI test. Additionally, each parent's age, gender, education, and former experience with anesthesia were recorded. Parents who were illiterate, refused to fill in the questionnaire form, had taken anxiolytic medications in the last 72 hours, or had hearing problems, psychiatric disorders, or senile dementia were not included in the study.

In the anesthesiology outpatient clinic, patients were randomly divided into two groups. In Group I, each patient's medical history and physical examination results were recorded in a standard patient file by an anesthesiologist. After informing the parents of each child of the risks of anesthesia, written consent for anesthesia was obtained from the parents. In Group II, after being routinely informed by the anesthesiologist, the parents of the patients received further detailed information (see the appendix) on safety measures taken during anesthesia; how the anesthesia would be administered; the drugs used and their possible side effects; possible complications of anesthesia and their interventions; and the postanesthesia period. The parents' questions were answered. Information was provided to all parents by the anesthesiologists responsible for the MRI and the anesthesia was performed by the same anesthesiologist. In all patients, induction of anesthesia was performed with midazolam (0.05 mg/kg) and propofol (0.5 mg/kg) and maintenance of anesthesia was ensured with propofol (1–3 mg/kg/h) with Ramsey sedation score being between 3 and 4. After the procedure, the followup and observation of the patients were done in a recovery room located in the radiology department; this room is particularly used for patients undergoing outpatient anesthesia. Patients could stay here with their parents.

Many test methods are used in the evaluation of anxiety scores [17–19]. In this study, after informing the parents about anesthesia, we used the State-Trait Anxiety Inventory (STAI) test developed by Spielberger and his coworkers in 1970. Oner and Le Compte have adapted the State-Trait Anxiety Inventory test into Turkish and have completed studies on its validity and reliability. Previous studies with control and patient groups have reported reliability coefficients between 83 and 87% for the STAI test [17].

For statistical analyses, the SPSS (Statistical Package for the Social Sciences) for Windows was used. The distributions of frequencies and mean values were calculated. For the comparison of categorical data, the chi-square test was used, and for the comparison of numerical data, Student's *t*-test was used. The relationship between the educational levels and the anxiety scores of the parents was evaluated using correlation analysis. A *P* value less than 0.05 was accepted as significant.

## 3. Results

The demographic data of the children are provided in Table 1. There was no significant difference between the two groups of children in terms of age, gender, weight, ASA scores, or the durations of their MRI tests. There was also no significant difference between the two groups in terms of the distribution of MRI tests of the brain and vertebra, the most frequently imaged organs (Table 1).

The demographic data of the parents are provided in Table 2. The state anxiety scores of the parents in Group I were significantly higher than those of the parents in Group II (mean scores: 43.61 ± 8.74 and 39 ± 9.82, resp., *P* = 0.003). Five patients in Group I and seven patients in Group II had had one or more previous experiences with anesthesia, and there was no significant difference between the two groups in terms of the number of former experiences (*P* = 0.93). However, in both groups, parents who had previously experienced anesthesia had lower anxiety scores than parents with no experience (Table 2). In both groups, there was a correlation between education levels and the anxiety scores of the parents (*P* = 0.020). Moreover, the anxiety scores of the parents with college degrees did not decrease when more detailed information was provided (Table 2).

## 4. Discussion

Children frequently experience fear, anger, anxiety, and a sense of guilt before an operation or any invasive intervention [20]. The child's age, the kind of disorder, the length of hospitalization, and the upcoming procedure all contribute to the child's anxiety, as well as to the anxiety of the parents. The parents' anxiety increases the child's and vice versa [21]. Lamontagne et al. found that children of parents with high anxiety scores at preoperation time also had high anxiety scores [22]. To decrease their anxiety, parents were allowed to stay with their children during the induction of anesthesia and were well-informed (using written, oral, and visual materials) of the details of the procedure [16].

Although practices differ between countries, allowing parents in the operation room during anesthesia induction is relatively common practice [23]. However, this practice has long been a matter of dispute [21]. Some authors have reported that allowing the parents to be present decreased the anxiety scores of both the children and their parents [24]. Other authors have claimed that the practice had no effect on anxiety [25]. In this study, we allowed parents to accompany their children until the intervention room, and parents were removed from the intervention room after induction of anesthesia.

Some studies have reported that the written information provided to children and their parents before anesthesia was both important and effective [26–29]. On the other hand, other studies have claimed that the preoperative information was insufficient [30]. In the literature, different methods were used for the purpose of the perioperative anesthetic informing of parents. Zuckerberg reported that informing parents about their child's disorder or the intervention to be performed decreased parents' anxiety [7]. Bellew et al. reported that written anesthesia information alone was superior to oral information in providing satisfactory information and decreasing anxiety scores [14]. Parents preferred to have complete perioperative information, although highly detailed information provided no change in anxiety score [10]. Some authors have claimed that providing detailed information only increased patient anxiety [31], while others have reported that providing detailed information was beneficial and anxiety levels did not increase [10, 14, 32]. Similarly, in this study, the state anxiety scores of the parents decreased significantly when the anesthesiologist provided detailed information about the procedure, in addition to the information form provided during the outpatient examination, before anesthesia. The decrease in anxiety could be due to increased trust in the doctor who provided the detailed information.

Oğuzalp et al. reported that the parents' educational levels had no effect on the relationship between the information provided prior to anesthesia and the parents' state anxiety scores [33]. On the contrary, Kain et al. reported that parents with higher educational levels had lower anxiety scores [34]. In our study, we observed that the parents' state anxiety scores increased in direct parallel with the parents' educational levels. In both groups, there was a significant correlation between educational levels and the state anxiety scores of the parents. When the two groups were compared, it was observed that detailed information decreased the anxiety scores of parents educated up to senior year of high school but caused no change in the scores of those with higher education levels.

Patient anxiety scores may change according to former experience with anesthesia [35, 36]. Badner et al. demonstrated that former anesthesia experience decreased patients' anxiety scores [37]. Similarly, in the present study, former anesthesia experience was associated with lower parental anxiety scores.

In previous studies, physiological (arterial blood tension and pulse) and neuroendocrinological (epinephrine, norepinephrine, and cortisol) parameters have been assessed to evaluate anxiety [38]. This study was limited in that parental anxiety was measured using only the State-Trait Anxiety Inventory.

In conclusion, informing parents in detail about interventions, in addition to providing routine outpatient information, before anesthesia may increase their trust in doctors and, thus, decrease their anxiety scores. However, further studies are required on the content of the information to be provided.

## Figures and Tables

**Table 1:** The children's parameters.

Parameters	Group I	Group II	*P*
Age (year)	3.4 ± 3.1	3.2 ± 2.8	*0.63 *
Gender			
Male *n* (%)	38 (48.7)	40 (51.3)	*0.87 *
Female *n* (%)	34 (50.0)	34 (50.0)	
Weight (kg)	15.6 ± 5.3	13.9 ± 6.2	*0.64 *
ASA I/II (*n*)	57/15	61/13	*0.61 *
Duration of MRI (min)	25.7 ± 10.4	24.6 ± 9.5	*0.72 *
MRI-body part imaged (*n*)			
Brain	35	40	*0.93 *
Columna vertebralis	20	21	
Abdomen	7	4	
Extremity-joint	3	2	
Spectroscopy	3	3	
Other	4	4	

The data are presented as mean ± SD. min: minutes; *n*: number; %: percentage.

ASA: American Association of Anesthesiologists Risk Classification.

**Table 2:** The parents' parameters.

	Group I	Group II	*P*
Age (year)	33 ± 7.8	32.5 ± 7.9	*0.70 *
Gender			
Male *n* (%)	31 (46.3)	36 (53.7)	*0.49 *
Female *n* (%)	41 (51.9)	38 (48.1)	
State anxiety score	43.6 ± 8.7	39.00 ± 9.82	*0.003 *
Former anesthesia experience			
Yes (*n*) STAI	(5) 39 ± 6.5*	(7) 33.3 ± 6.5*	***0.58***
No (*n*) STAI	(67) 47.4 ± 8.9	(67) 39.1 ± 6.5	
The state anxiety scores according to the scanned body region			
Brain (*n*) STAI	(35) 42.37 ± 8.4	(40) 40.23 ± 9.6	*0.30 *
Columna vertebralis (*n*) STAI	(20) 43.05 ± 9.2	(21) 38.7 ± 10.1	*0.16 *
The state anxiety scores according to educational level			
Primary school (*n*) STAI	(23) 38.01 ± 4.32	(28) 32.59 ± 6.12	***0.048***
Junior-senior high (*n*) STAI	(38) 41.40 ± 2.28	(33) 36.33 ± 3.78	***0.050***
University (*n*) STAI	(11) 50.20 ± 2.04	(13) 48.18 ± 2.54	*0.381 *

The data are presented as mean ± SD. *n*: number; STAI: State-Trait Anxiety Inventory.

*Significant within the group.

## Appendix

### The Detailed Information Form

Why is anesthesia required during MRI?

The environment is very noisy during the MRI scanning process and also children may be very worried when separated from their families for the scan. To avoid the child's possible anxiety and to ensure the image quality, it is necessary to use anesthesia.

How to carry out anesthesia?

In order to ensure that the child remains motionless during MRI “anesthetic” agents with soporific effects are used. These drugs are administered intravenously. An intravenous needle needs to be inserted first to give your child intravenous fluids and medications.

What does the anesthesiologist do?

Before the procedure, the anesthetist examines your child's health status and selects the most appropriate anesthesia. During the MRI, the anesthesiologist is always located next to your child. Intravenous anesthetic drugs with calming effects are given to your child during the procedure to ensure that the child does not worry and also to prevent the movement of the child. During this time, the anesthesiologist monitors the heart rate, blood pressure, oxygen saturation, and respiratory functions constantly. They are also responsible for putting the patient to sleep safely and waking the patient after the procedure just as safely. These measurements of your child's vital signs through the process are essential to ensure adequate and safe depth of anesthesia.

Does anesthesia have risks?

Although the methods used for anesthesia today are very advanced with the use of sophisticated monitors and very safe drugs, they are not completely risk-free. With the development of technology and medicine, complication rates have fallen considerably, but still some complications may occur due to unforeseen factors.

Depending on the drugs used and the anesthetic effect, non-life-threatening nausea, sore throat, nonserious allergic reactions (itching, redness, etc.), chills, and rigors can be frequently seen. Although they are rather rare, much more serious complications such as the fact that there could be adverse effects on the heart function, the pulse, and the blood pressure may drop to low levels; if the respiratory functions are not monitored sufficiently, there could be lack of oxygen, the patient may vomit during the procedure while under sedation, also very rarely (1/10.000 patients) the contents of the stomach may seep into the lungs, severe allergic reaction to the medication or the radiopaque substance may develop, and death (1/200.000) may occur. As the anesthesia team, we take every precaution to avoid such serious complications; however, if an unexpected complication develops, we have the equipment and the experience to make necessary interventions.

When do the effects of anesthesia cease?

After the MR procedure is over, drug administration will be stopped and your child's vital signs will be monitored. Until the patient is fully awake and the vital signs are stable, the patient will be monitored constantly in the recovery room. This process may take approximately 30–45 minutes. However, even if your child is released from the hospital, there should be an adult supervisor with the child and for at least 2 hours the child must not be left alone, he/she should rest, and he/she should be prevented from eating and drinking.
